# Free-running time-resolved first-pass myocardial perfusion using a multi-scale dynamics decomposition: CMR-MOTUS

**DOI:** 10.1007/s10334-025-01291-x

**Published:** 2025-09-23

**Authors:** Thomas E. Olausson, Maarten L. Terpstra, Niek R. F. Huttinga, Casper Beijst, Niels Blanken, Dominika Suchá, Teresa Correia, Birgitta K. Velthuis, Cornelis A. T. van den Berg, Alessandro Sbrizzi

**Affiliations:** 1 Computational Imaging Group for MR Therapy and Diagnostics, University Medical Center Utrecht, Utrecht, Netherlands; 2 Department of Radiotherapy, UMC Utrecht Cancer Center, University Medical Center Utrecht, Utrecht, Netherlands; 3 Department of Radiology, University Medical Center Utrecht, Utrecht, Netherlands; 4 School of Biomedical Engineering and Imaging Sciences, King’s College London, London, UK; 5 Centre for Marine Sciences, Faro, Portugal

## Abstract

**Objective:**

First-pass myocardial perfusion involves several types of dynamics, including cardiac motion, respiratory motion, bulk motion and contrast agent inflow. To accurately quantify the initial inflow of the contrast agent, high spatiotemporal resolution MR imaging must be obtained. To achieve this, we present a novel approach, named CMR-MOTUS, for the reconstruction of time-resolved free-running first-pass myocardial perfusion by jointly estimating high-quality motion fields and contrast-varying images.

**Materials and methods:**

We propose CMR-MOTUS, which extends the MR-MOTUS framework by integrating a contrast-varying reference image with a low-rank plus sparse decomposition to capture additional dynamics such as blood flow and contrast agent inflow. This joint reconstruction framework alternates between solving for time-dependent image contrast changes and motion fields, eliminating the need for a pre-acquisition motion-static reference image. The method was tested on simulations and in-vivo datasets.

**Results:**

In simulations, CMR-MOTUS showed improved image similarity and motion field accuracy compared to state-of-the-art methods. In in-vivo tests, the methods effectively captured cardiac and respiratory motion dynamics, resulting in cine images with sharper features than state-of-the-art.

**Discussion:**

CMR-MOTUS presents significant advantages by modelling motion and contrast dynamics in the reconstruction of first-pass myocardial perfusion. The framework enables a data-efficient free-running workflow since the entire acquisition is correlated with high-quality motion fields. This approach has the potential to enhance the diagnostic value of cardiac MRI but needs further clinical validations.

**Supplementary Information:**

The online version contains supplementary material available at 10.1007/s10334-025-01291-x.

## Introduction

First-pass myocardial perfusion with MRI is primarily used to detect ischemia in patients with (suspected) coronary artery disease (CAD) [1, 2]. These examinations involve a continuous acquisition of at least 60 s spanning several types of dynamics, including cardiac motion, respiratory motion, bulk motion, and gadolinium-based contrast agent (GBCA) inflow. To accurately quantify the initial inflow of the GBCA, high spatiotemporal resolution imaging must be obtained during this narrow temporal window, which makes the examination time-sensitive. Hence, the motion, anatomy, and contrast dynamics need to be separated when reconstructing time-resolved MRI to have an accurate first-pass myocardial perfusion quantification. Additionally, the extracted motion dynamics can be used for explicit motion quantification, which has clear benefits: high-quality motion fields are diagnostically valuable as they can be used in further image analysis, such as characterizing myocardial strain [3]. Therefore, efficient time-resolved reconstruction methods that enable the separation of motion and contrast dynamics are needed for accurate first-pass myocardial perfusion.

In cardiovascular MRI (CMR), numerous studies have focused on enhancing the efficiency and accuracy of image reconstruction by addressing both cardiac and respiratory motions within a free-running CMR protocol [4, 5]. These methods also work well when incorporating explicit signal evolution knowledge, such as for quantitative T1/T2 mapping MRI [5]. However, modelling the signal evolution of first-pass myocardial perfusion from the GBCA in the image reconstruction is challenging. Recently developed methods attempted to resolve bulk and respiratory motion using Robust principal component analysis (RPCA) [6] techniques on electrocardiogram (ECG)-triggered first-pass myocardial perfusion to disentangle the contrast enhancement from the registration [7, 8]. Other methods have looked into ECG-free first-pass myocardial perfusion examinations by extracting a surrogate signal for the cardiac phase during a continuous acquisition followed by sorting the data based on the surrogate signal before motion correcting for the respiratory and bulk motion in a cardiac phase [9]. However, these registration algorithms struggle with the strong intensity variations during the GBCA inflow, which make cardiac motion correction difficult [10]. Finally, methods that rely on sorting the data into cardiac and respiratory phases or triggering are inherently time-inefficient and sensitive to bulk motion, which can corrupt the time-sensitive inflow of the contrast agent [11].

To use all the data efficiently and reconstruct time-resolved MRI, researchers have focused on integrating powerful methods from video processing, such as the low-rank plus sparse decomposition (L + S) [12, 13]. The low-rank (L) component generally captures changes in larger structures that are present, while the sparse (S) component usually captures the small-scale intensity variations, such as the enhancement of vessels [12]. Overall, L + S is a flexible regularization method representing spatiotemporal dynamics using a few principal components, constraining the solution space. However, accurately capturing complex dynamics requires a large number of components, limiting the effectiveness of this method. Alternatively, motion dynamics are more compressible with respect to degrees of freedom than image dynamics and better suited for these applications [14]. Additionally, the inclusion of motion fields into the image reconstruction problem has been shown to reduce blurring in motion-guided L + S reconstructions [15, 16]. Hence, this motivates the need to extract high-quality time-resolved motion fields.

One method explicitly relating the k-space data of frames to low-dimensional motion fields of a high spatial resolution motion-static reference image, named MR-MOTUS, has demonstrated promising results in extracting high-quality breathing motion fields [17, 18]. This approach facilitates time-resolved reconstructions with high temporal resolutions of 100 ms in free-running 3D cine MRI by separating respiratory and bulk motion [17]. MR-MOTUS’ success relies on the assumption that a dynamic object in an MRI scanner can be described as a single, static reference image that is deformed by low-dimensional motion fields, implying the conservation of magnetization during deformation. This assumption is valid in abdominal imaging, but is not valid in cardiac imaging as blood and GBCA flow in and out of the field of view. Another technical drawback of MR-MOTUS is that it requires a motion state reference image before the motion estimation step. Obtaining a reference image may not always be feasible or easily accessible. While breath-holds or gating techniques can be used to obtain these reference images [18], they can prolong the examination time and pose extra challenges for clinicians.

In this work, we investigate the reconstruction of time-resolved free-running first-pass myocardial perfusion MRI using a Contrast-varying Model-based Reconstruction of Motion from Undersampled Signal (CMR-MOTUS) that extends the original MR-MOTUS [17, 18, 20] framework by reconstructing, in addition to the motion fields, an L + S [12, 16] image series. Cardiac, respiratory, and bulk motion dynamics are extracted using MR-MOTUS, whilst the GBCA dynamics are captured using an L + S decomposition. Thus, we efficiently disentangle the different types of dynamics during a time-resolved free-running first-pass myocardial perfusion acquisition from patient datasets. This approach offers significant advantages by reducing examination time and complexity while providing high-quality motion fields and motion-corrected perfusion image series.

## Theory

In a conventional MR-MOTUS framework, we define target time-series images$H = [h_{1} ,...,h_{M} ]$ a motion static reference image in its vectorized form ${\boldsymbol{q}}$ of length $N$ (total number of voxels) and motion fields $D = [D_{1} ,...,D_{M} ]$ for each dynamic index $t$, up to $M$ total number of dynamics. These quantities are mutually related as follows,

1 $$h_{t} (r) = q(D_{t} (r)) \cdot \det (\nabla D_{t} )$$

where ${\boldsymbol{r}}=\left(x,y,z\right)$ are spatial coordinates and $\nabla$ is the Jacobian operator. From Eq. (1), ${{\boldsymbol{h}}}_{t}$ is approximated by warping the reference image ${\boldsymbol{q}}$, which is the fundamental requirement for the MR-MOTUS framework. This approximation is incorporated into the MR signal model leading to the following MR-MOTUS signal model ${\hat{\mathrm{F}}}$ [18].

2 $$S_{t} (k) = \hat{F}(D_{t} |q) = \int_{\Omega } {q(r)} \,e^{{ - i2\pi k \cdot D_{t}^{ - 1} (r)}} dr$$

The notation $\hat{F}(D_{t} |q)$ denotes that the model is conditional to the specific value of ${\boldsymbol{q}}$. Here, the reference image ${\boldsymbol{q}}$ is related to the motion fields ${\boldsymbol{D}}$ at time point $t$ for the measured k-space data $s_{t}$, and ${\boldsymbol{k}}=\left({k}_{x},{k}_{y},{k}_{z}\right)$ are k-space coordinates.

One limitation in the previous MR-MOTUS implementations, the coil dimension of the k-space has been compressed into a single homogeneous coil to reduce computation time. In this paper, we assume that the coil maps are static throughout the acquisition. Meaning that a single reference image needs to be warped before coil maps are applied.

Another limitation is that it does not consider contrast variation in the reference image, which can occur in cardiac examinations due to blood flow or contrast administration. Additionally, ${\boldsymbol{q}}$ must be motion static, meaning that breath-hold or gated images need to be acquired in a preparation phase before the examination to use the MR-MOTUS framework. To tackle these challenges, we made a small but crucial extension to the MR-MOTUS framework where the reference image is allowed to change over time, resulting in a time series of images $Q = [q_{1} ,...,q_{M} ]$. Although this modification enhances the validity and flexibility of the MR-MOTUS model, it also drastically augments the number of unknowns in the reconstruction problem, making it even more ill-posed and challenging to solve for the motion fields. To implement this, we use the low-rank plus sparse (L + S) decomposition [12] as a regularizer for ${\boldsymbol{Q}}$. Additionally, there is no longer a need for a preparation phase to acquire a motion static reference image, instead contrast-varying motion static reference images are jointly estimated during alternating iterations using the entire time-resolved data. We call this joint approach CMR-MOTUS.

According to this approach, H is approximated by

3 $$h_{t} (r) = q_{t} (D_{t} (r)) \cdot \det (\nabla D_{t} )$$

Hence, a slightly modified signal model F is derived to include this time-varying reference image Q

4 $$S_{t} (k) = \hat{F}(D_{t} |q) = \int_{\Omega } {q(r)} e^{{ - i2\pi k \cdot D_{t}^{{ - 1}} (r)}} dr dr$$

The goal is to gradually disentangle the motion and contrast variations by alternating between the L + S decompositions and the MR-MOTUS framework.

In summary, CMR-MOTUS relies on the alternating solution of two reconstruction problems, namely:

1. Image reconstruction to determine the time-dependent image contrast changes $Q$.
2. Motion estimation to determine the motion fields $D$.

We briefly describe both alternating steps.

### Image reconstruction

In the literature of L + S decomposition in time-resolved MRI [12], the entire target time-series images ${\boldsymbol{H}}$ is approximated by a low-rank term ${\boldsymbol{L}}$ plus a sparse term ${\boldsymbol{S}}$. The ${\boldsymbol{L}}$ term captures the larger scale slow changes in the time series, while the ${\boldsymbol{S}}$ term captures the finer details in the series, including the contrast enhancement [12].

In this work, we instead leverage motion fields using MR-MOTUS to motion correct the L+S reconstruction process. This is achieved by using the CMR-MOTUS model presented in eq (4) in the data consistency term of the original L+S inverse problem [12].

5 $$[L^{*} ,S^{*} ] = \arg \min _{{L,S}} \sum\limits_{t}^{M} {\left\| {F(q_{t} |D_{t} ) - s_{t} } \right\|_{2}^{2} } + \lambda _{L} \left\| L \right\|_{*} + \lambda _{s} \left\| {TS} \right\|_{1} {\mathrm{with}} Q = L + S$$

where ${\boldsymbol{L}}+{\boldsymbol{S}}$ now approximates the time-varying reference image ${\boldsymbol{Q}}$, and the Fourier transform now contains the time-dependent motion fields as described by $\mathbf{F}$. Note that the variables in the notation $F(q_{t} |D_{t} )$ are switched, now meaning that the model is conditional on the specific value of ${\boldsymbol{D}}$. $\left\| {{\phantom{a}}} \right\|_{*}$ denotes the nuclear norm, $\left\| {{\phantom{a}}} \right\|_{1}$ the L1-norm, and ${\uplambda }_{\mathrm{L}}$ and ${\uplambda }_{\mathrm{S}}$ are the weights for the regularization terms involving ${\boldsymbol{L}}$ and ${\boldsymbol{S}}$, respectively. Additionally, we take advantage of sparsity in the temporal Fourier domain using the temporal Fourier operator $\mathcal{T}$, as it has shown promising results in first-pass myocardial perfusion applications[12].

Technically, we have the flexibility to designate any motion state at time $t$ as a reference motion static state by subtracting its influence on all the other motion fields. For instance, the reference static phase could correspond to the diastolic one. This adjustment is done before solving Eq. 6 and it steers the L + S reconstruction process towards a diastolic reference motion static state. In this work, we opt to reconstruct a mid-position ${\boldsymbol{Q}}$ by subtracting the temporal mean of the motion fields from ${\boldsymbol{D}}$.

Since we use a non-differentiable regularizer, we use a custom proximal gradient method based on the Fast Ite (rative/Shrinkage Thresholding Algorithm (FISTA) [21, 22] to solve equation (6).

### Motion estimation

As described in Huttinga et al. [17], non-rigid motion fields in MRI can be decomposed into a low-rank model with principal components$\Phi$ and $\Psi$. For an explicit Rank $R$,$\Phi$ is a matrix with size $2N \times R$ ($3N \times R$for 3D), describing the spatial components and $\Psi$is a matrix with size $M\times R$ describing the temporal components. The full motion fields are then approximated by $D \approx \Phi \Psi^{{\mathrm{T}}}$ . Additionally, we parameterize the low-rank motion fields using a B-spline basis in space and time as described by Huttinga et al. [17]. Hence, we can solve the following inverse problem for an explicit low-rank representation of the motion fields, similar to the low-rank MR-MOTUS method, but using a time-varying contrast image q_t_ rather than a fixed reference:

6 $$[\Phi^{*} ,\Psi^{*} ] = \arg \,\min_{{\Phi \Psi^{T} }} \sum\limits_{t}^{M} {\left\| {F(D_{t} \left| {q_{t} ) - s_{t} \left\| {_{2}^{2} } \right.} \right.} \right.} + \left. {\lambda_{TV} } \right\|TV\left. {(D)} \right\|_{2,1}$$

The 2, 1 norm refers to the “mixed norm” of the isotropic total variation TV as defined in [19, 23]

7 $$TV: = \sqrt {\left( {\sum\nolimits_{i}^{N} {\left\| {[\nabla D_{t}^{x} ]_{i} } \right\|_{2} } } \right)^{2} + \left( {\sum\nolimits_{i}^{N} {\left\| {[\nabla D_{t}^{y} ]_{i} } \right\|_{2} } } \right)^{2} }$$

We use the L-BFGS [24] algorithm to solve equation (6^7^).

**Fig. 1:**
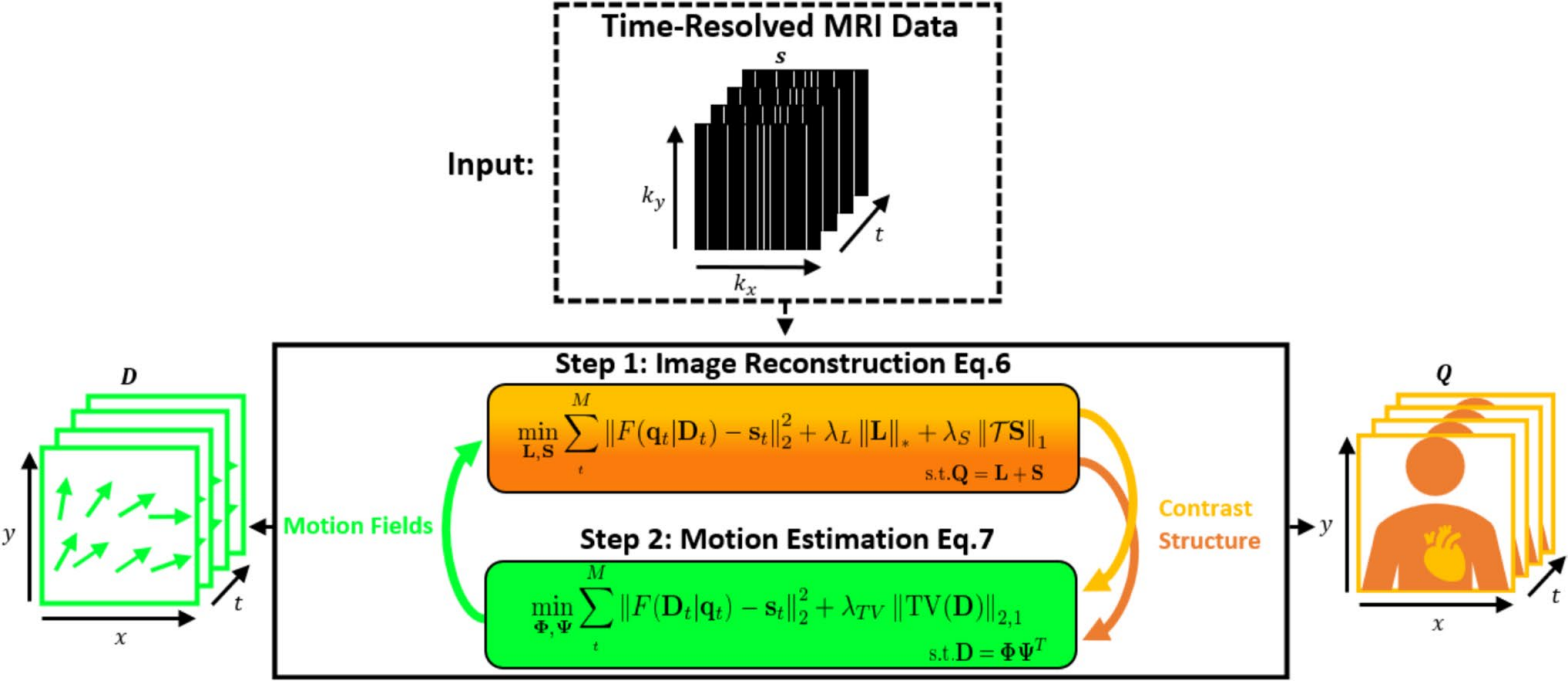
Schematic representation of the proposed CMR-MOTUS framework. The framework takes raw time-resolved $t$ MRI data ${s}_{t}$. In step 1, the contrast-varying reference images ${q}_{t}$ are solved while the motion fields $D$ are fixed, using an L + S reconstruction. In step 2, the motion fields $D$ are solved while the contrast-varying reference images $q$ are fixed. The output of step 1, shown on the right, includes the contrast component $S$ and structure component $L$. Step 1 and step 2 are solved iteratively in an alternating manner until the motion and contrast are disentangled. The output of step 2, shown on the left, includes the motion fields $D$

Figure 1 summarizes the CMR-MOTUS framework. The iterative process alternates between steps 1 and 2 until the time-varying reference image of the motion state is accurately captured in step 1 and the motion dynamics are effectively captured by the motion fields in step 2. The optimal regularization weights ${\uplambda }_{{\mathrm{L}}}$, ${\uplambda }_{{\mathrm{S}}}$, ${\uplambda }_{{{\mathrm{TV}}}}$, and the maximum number of alternations and inner iterations are determined empirically (see Methods section).

## Methods

Here we give a brief overview of all experiments, and further in-depth experimental details are described later in the methods. First, we tested our proposed CMR-MOTUS method on first-pass myocardial perfusion simulations using the MRXCAT [25] framework. This was to show that we achieved similar image quality to our motion-resolved cines compared to state-of-the-art L + S image reconstructions with similar settings, whilst extracting diagnostically valuable motion fields.

Subsequently, we demonstrated the feasibility of the proposed method on two publicly available datasets of time-resolved cardiac cine MRI without first-pass perfusion taken from the OCMR dataset [26]. They provided in-vivo prospectively undersampled raw cartesian multi-coil k-space CINE data, which were acquired during free-breathing and without ECG-triggering. This data was affected by breathing motion, cardiac motion, and blood flow dynamics, which we resolved in the proposed reconstruction. Since ground truth reconstructions are not available, we used the Tenengrad function [27, 28], a gradient-based reference-free sharpness metric, to show the improved reconstruction quality when modelling motion with motion fields as in CMR-MOTUS.

Finally, we used two radially acquired first-pass myocardial perfusion MRI datasets of patients scheduled for a cardiac MRI examination to analyze the proposed CMR-MOTUS reconstruction method. The first dataset was acquired with ECG-triggering and a breath hold, whilst the other dataset was acquired without ECG-triggering and in free breathing. These first-pass myocardial perfusion reconstructions highlighted the ability of the proposed framework to separate the physiological motion dynamics from the contrast agent inflow.

Additionally, we highlighted the flexibility of the framework to non-cartesian and cartesian sampling schemes.

### Implementation

The proposed alternating reconstruction was a custom extension to the low-rank MR-MOTUS [17] framework, together with an in-house L + S decomposition implementation in MATLAB. All reconstructions are performed on the CPU of a computational server (AMD Ryzen Threadripper 3970X 32-Core 3700 MHz and 256 GB RAM). The number of alternating steps was decided empirically (convergence was usually observed after 5 alternating steps), with a maximum number of inner iterations set to be 60 in step 2, and step 1 was set to stop if the normalized difference in the total objective value was below $1 \cdot 10^{ - 4}$ over 4 consecutive iterations. Step 1 usually converged within 10 iterations.

The entire proposed alternating framework, which is illustrated in Fig. 1, was initialized with a time-averaged reference image generated by step 1 with $\mathbf{D}=0$. Step 2 began with this reference image. At each solution of step 2, $\Phi$ and $\Psi$ were initialized with random numbers between $-1$ and $1$. Whilst in step 1, the L + S reconstructions were initialized with the previous estimations of the $\mathbf{L}$ and $\mathbf{S}$ components. The step size for step 2 was determined to be ${{1.3} \mathord{\left/ {\vphantom {{1.3} \gamma }} \right. \kern-0pt} \gamma }$ [22], where $\gamma$ is the largest eigenvalue of ${\boldsymbol{F}}{{\boldsymbol{F}}}^{*}$ which was determined via a power method [29].

### Test 1: in silico

The XCAT phantom was used to generate six hundred frames with cardiac motion at a temporal resolution of 50 ms and a matrix size 100 × 100. The orientation was set to a short-axis view of the heart. The heart cycle period was set to 1 s. With these settings, XCAT provided ground truth (GT) segmentations and motion fields for each time frame.

A saturated-recovery gradient echo sequence of first-pass myocardial perfusion was simulated resulting in coil-sensitive MR-contrast XCAT frames using the MRXCAT framework [25]. The acquisition parameters were $T_{R} = 2.0\,{\mathrm{ms}}$, $T_{sat} = 150\,{\mathrm{ms}}$, ${\mathrm{flip}}\,{\mathrm{angle}} = 15^\circ$, and 8 number of coils. We simulated undersampling by multiplying a sampling mask with the Fourier transform of each frame. The sampling mask followed a pseudo-random Cartesian sampling pattern [30] with $12$ readouts per frame, corresponding to an acceleration factor of 10. Coil maps were estimated using ESPIRIT [31]

### Reconstructions

- Proposed (CMR-MOTUS): We performed the proposed alternating reconstructions on the in silico data with ${\uplambda }_{s} = 10$ and explicitly enforce rank one for both the motion fields and the images $\mathrm{L}$ component with the aim to reconstruct only cardiac motion fields, a static single frame low-rank image, and sparse images that capture the contrast enhancement. The motion model used 80 b-spline control points in each spatial direction. Regularization weights were empirically set to ${\uplambda }_{TV} = 1 \cdot 10^{ - 8}$.
- L + S: For comparison purposes, we reconstruct L + S images with the same settings as used with the proposed, so that we obtain non-motion-corrected images. The motion fields of the L + S reconstruction were separately determined for each reconstructed frame with the Elastix [32] registration toolbox using the first frame as the fixed image.

### Quantitative validations

- Image quality: The mean structural similarity index (SSIM) [33] with respect to the ground truth MRXCAT frames was determined for the CMR-MOTUS time-series images $H$, and the L + S reconstruction.
- Motion fields: The end point error (EPE) with respect to ground truth XCAT motion fields over the entire field of view (FOV) was determined for the proposed MR-MOTUS motion fields, and the motion fields estimated with Elastix after L + S reconstruction. The EPE was determined by$EPE = \sqrt {\left( {D_{x,predicted} - D_{x,ground\,truth} } \right)^{2} + \left( {D_{y,predicted} - D_{y,ground\,truth} } \right)^{2} }$ To evaluate the accuracy of the motion fields over the myocardium, the first segmentation of the XCAT series was warped according to both methods, which resulted in two time-resolved segmentations. The DICE score with the XCAT segmentations as a reference was determined for the warped first frame XCAT segmentation of the myocardium with the estimated motion fields from MR-MOTUS and Elastix, respectively.

### Test 2: in-vivo free-running time-resolved CMR

Two publicly available multi-coil k-space datasets of prospectively cartesian undersampled 2D free-breathing balanced steady-state free precession (bSFFP) single-slice cardiac MRI cine series in a short axis (SAX) and long axis (LAX) view were used in the analysis [26]. Both datasets were reconstructed using the proposed CMR-MOTUS reconstruction, and a compressed sensing (CS) reconstruction using the BART [34] toolbox. The datasets acquired 3 and 6 consecutive heart cycles during free-breathing and are time-resolved which resulted in 65 and 128 temporal frames, respectively. Coil maps were estimated on 8 virtual coils with ESPIRIT. We refer the reader to the reference for more details on the acquisition [26].

For the proposed CMR-MOTUS reconstruction, we explicitly modelled one principal component (Rank = 1) in the L images.${\uplambda }_{s} = 1$. Since breathing and cardiac motion are expected, the motion model was explicitly defined to use 4 components (Rank = 4). This allowed us to capture these motion dynamics, including some additional flexibility to either capture other types of motion or enhance the deblurring of the initial reference image. To include smoothness priors, the motion model used 90 b-spline control points in each spatial direction and 40 in the temporal domain. The specific numbers of control points was chosen to allow flexibility without over-smoothing and restricting the motion fields. The regularization parameter were empirically set to ${\uplambda }_{TV} = 1 \cdot 10^{ - 8}$. For comparison, the CS reconstruction was performed using spatial wavelet and temporal total variation regularizations whose weights were empirically tuned. Since Ground Truth data is not available, the Tenengrad sharpness metric was determined to evaluate image quality for the proposed CMR-MOTUS time-series images H and the CS reconstruction.

### Test 3: in-vivo breath-hold ECG-triggered first-pass myocardial perfusion

One patient dataset [35] of an ECG-triggered saturation-recovery TFE sequence during first-pass myocardial perfusion was used in the analysis to showcase bulk motion correction. The data was acquired on a Philips 3T Achieva scanner. In-plane resolution was 1.6×1.6 mm^2^, FOV = 320×320 mm^2^, slice thickness =10 mm, 32 coils, and a total acquisition time of 1 min. A radial sampling scheme was used with 10 spokes per frame. The k-space was gridded to a Cartesian grid. Coil maps were estimated using ESPIRIT. The patient’s breath-hold was imperfect resulting in bulk motion as the patient inhaled again. This dataset was used to showcase how the proposed CMR-MOTUS framework will perform with imperfect breath-holding during first-pass myocardial perfusion. We refer the reader to the reference [35] for more details on the acquisition.

A L + S (Eq. 5) and CMR-MOTUS reconstruction were performed to visually compare the results of no motion correction and motion correction. For both reconstructions,${\uplambda }_{s} = 1$ and we explicitly model one component in the L images. The motion model was explicitly defined to use one component and used 80 B-spline control points in each spatial direction. No B-spline parameterization of the temporal component was used since temporally non-smooth motion is expected. Motion field regularization was decided empirically and set to${\uplambda }_{TV} = 1 \cdot 10^{ - 7}$.

### Test 4: in-vivo free-running time-resolved first-pass myocardial perfusion

Another patient dataset of a free-breathing saturation-recovery bSFFP sequence during first-pass myocardial perfusion without any ECG-triggering was used in the analysis. No patient information was required as only anonymized raw k-space data was used. This data was used to showcase how the proposed CMR-MOTUS framework will perform with contrast enhancement, respiratory motion and cardiac motion. The data was acquired on a Philips 1.5 T Ingenia scanner. Relevant acquisition parameters include an in-plane resolution of $2.73 \times 2.73\,{\mathrm{mm}}^{2}$, FOV = 350×350 mm^2^, slice thickness =10 mm, 16 coils, TE/TR$= {{1.29} \mathord{\left/ {\vphantom {{1.29} {2.6}}} \right. \kern-0pt} {2.6}}\,{\mathrm{ms}}$ , flip angle= 50°, saturation delay =100 ms, and a total scan time of $90 {\mathrm{s}}$. A pseudo golden angle radial sampling scheme was used with 20 spokes per frame. The k-space was gridded to a Cartesian grid. Coil maps were estimated using ESPIRIT.

A L + S and the proposed CMR-MOTUS reconstruction were performed to visually compare the results of no motion correction and motion correction. In both frameworks, the S component of the images regularization was set to ${\uplambda }_{s} = 5$ and the L component of the image was explicitly set to solve for one component (Rank = 1). The motion model was explicitly defined to use three components (Rank = 3). Given the anticipated cardiac and respiratory motion in the dataset, two components were allocated to capture each type of motion, with an additional component providing extra flexibility. To include some smoothness, the motion fields used 64 B-spline control points in each spatial direction and 150 in the temporal domain for 800 temporal frames. Regularization parameters were determined empirically and set to${\uplambda }_{TV} = 1 \cdot 10^{ - 8}$.

## Results

### Test 1: in silico

**Fig. 2:**
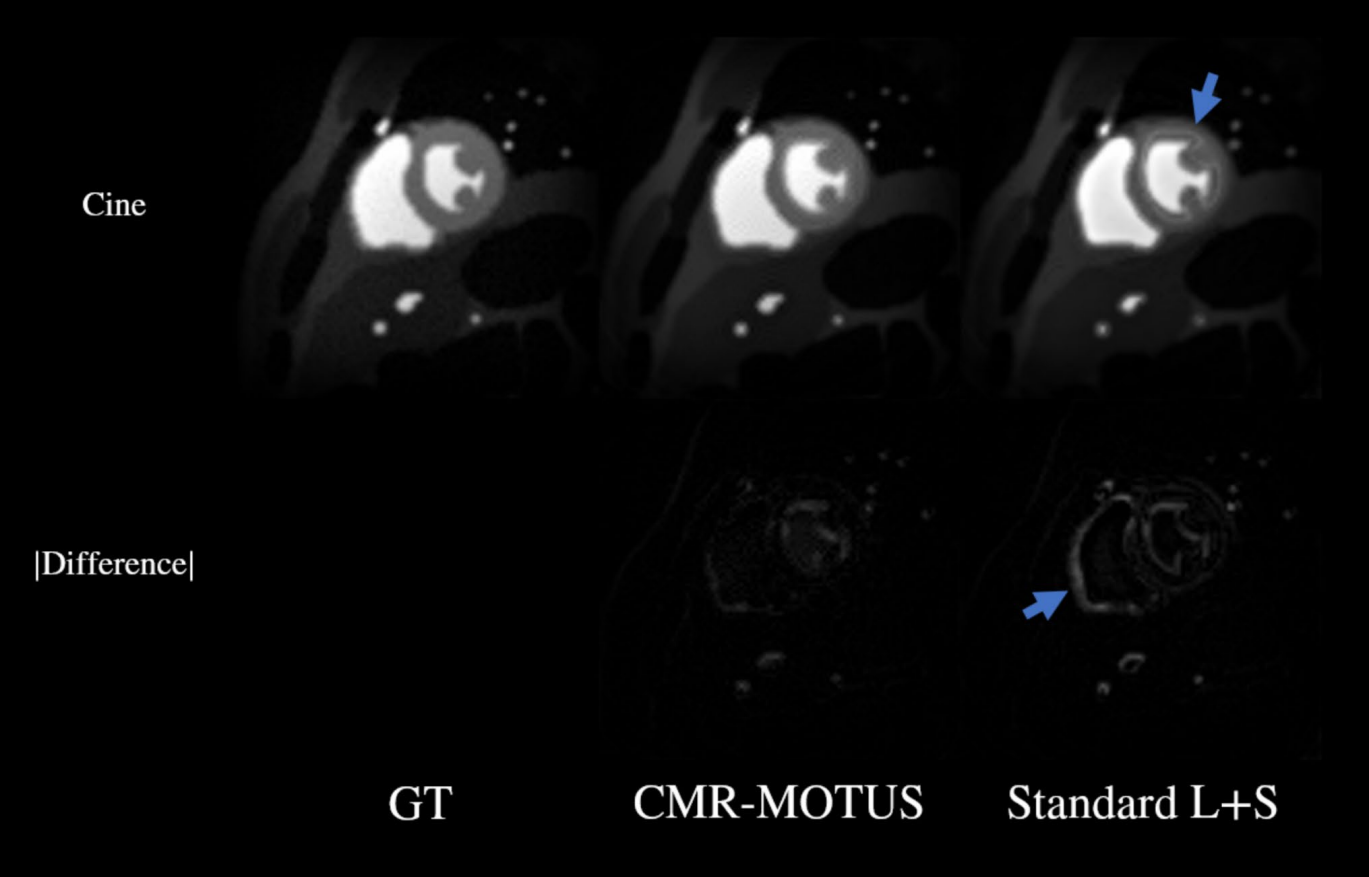
An end-systole frame during contrast agent inflow from the cine reconstructions using data from experiment 1. Left: the ground truth (GT) MRXCAT first-pass myocardial perfusion frames. Center: the cine reconstructed using CMR-MOTUS. Right: the cine reconstructed using a standard low-rank plus sparse (L + S) decomposition

A still frame from the reconstructed cines during an end diastole and contrast inflow is presented in Fig. 2. There is less temporal blurring in the CMR-MOTUS cine than in the standard L + S cine as indicated with the blue arrows. This is most apparent where the LV does not contract in the standard L + S cine and the difference is relatively large. Using the MRXCAT cine frames as a reference, the CMR-MOTUS cine and L + S cine resulted in a temporal mean SSIM value of $0.9780$ ($SD=0.0145$) and $0.9706$ ($SD=0.0155$) over the myocardium respectively, Wilcoxon signed-ranked test $p<0.001$. Thus, this indicates an improved image similarity with the inclusion of motion fields in CMR-MOTUS cine. The warped initial myocardium segmentation with the estimated motion fields resulted in a temporal mean DICE score of $0.9117$ ($SD=0.0624$) using CMR-MOTUS and $0.8913$ ($SD=0.0553$) using L + S and Elastix method, respectively, Wilcoxon signed rank test $p<0.001$. Hence, in this test CMR-MOTUS was able to reconstruct more accurate images as well motion fields w.r.t. a L + S reconstruction with the same settings.

**Fig. 3:**
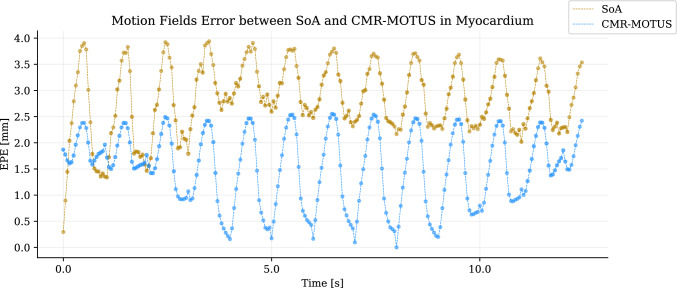
v Data from experiment 1 shows the mean endpoint error (EPE) in millimeters as a function of time for the estimated motion fields, using the XCAT motion fields as a reference over the entire field of view (FOV). The motion fields were estimated using a combination of low-rank plus sparse (L + S) decomposition image reconstruction followed by Elastix image registration (SoA, gold line), and the CMR-MOTUS framework (blue line). The plot reveals a clear lower mean EPE over time using CMR-MOTUS compared to the SoA motion-field reconstruction

Figure 3 showcases the time-resolved EPE of the motion fields estimated with each method with respect to the GT XCAT motion fields over a segmentation of the myocardium. CMR-MOTUS motion fields result in a lower EPE compared to motion fields estimated by Elastix.

### Test 2: in-vivo time-resolved CMR

**Fig. 4:**
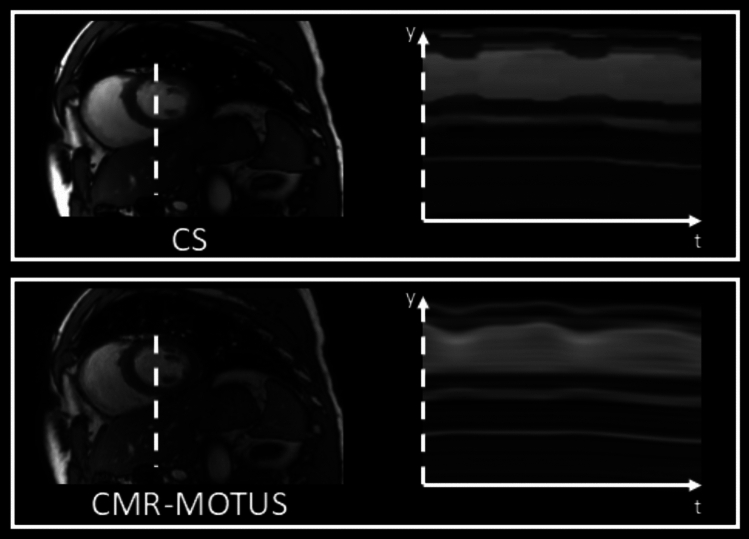
Frame from the reconstructed cine of the short axis (SAX) views in experiment 2 using compressed sensing (CS) on and CMR-MOTUS. y–t profile plots are shown beside each image. CMR-MOTUS shows a smooth motion profile, while some regions in the CS are blocky and motion static

The reconstructed SAX cine images are shown in Fig. 4. Both methods capture the entire cardiac and respiratory motion dynamics during the acquisition. The mean Tenengrad metric of the CMR-MOTUS cine was 14% larger than the CS cine, Wilcoxon signed-ranked test $p<0.001$, which indicates a sharper cine is reconstructed when using CMR-MOTUS.

**Fig. 5:**
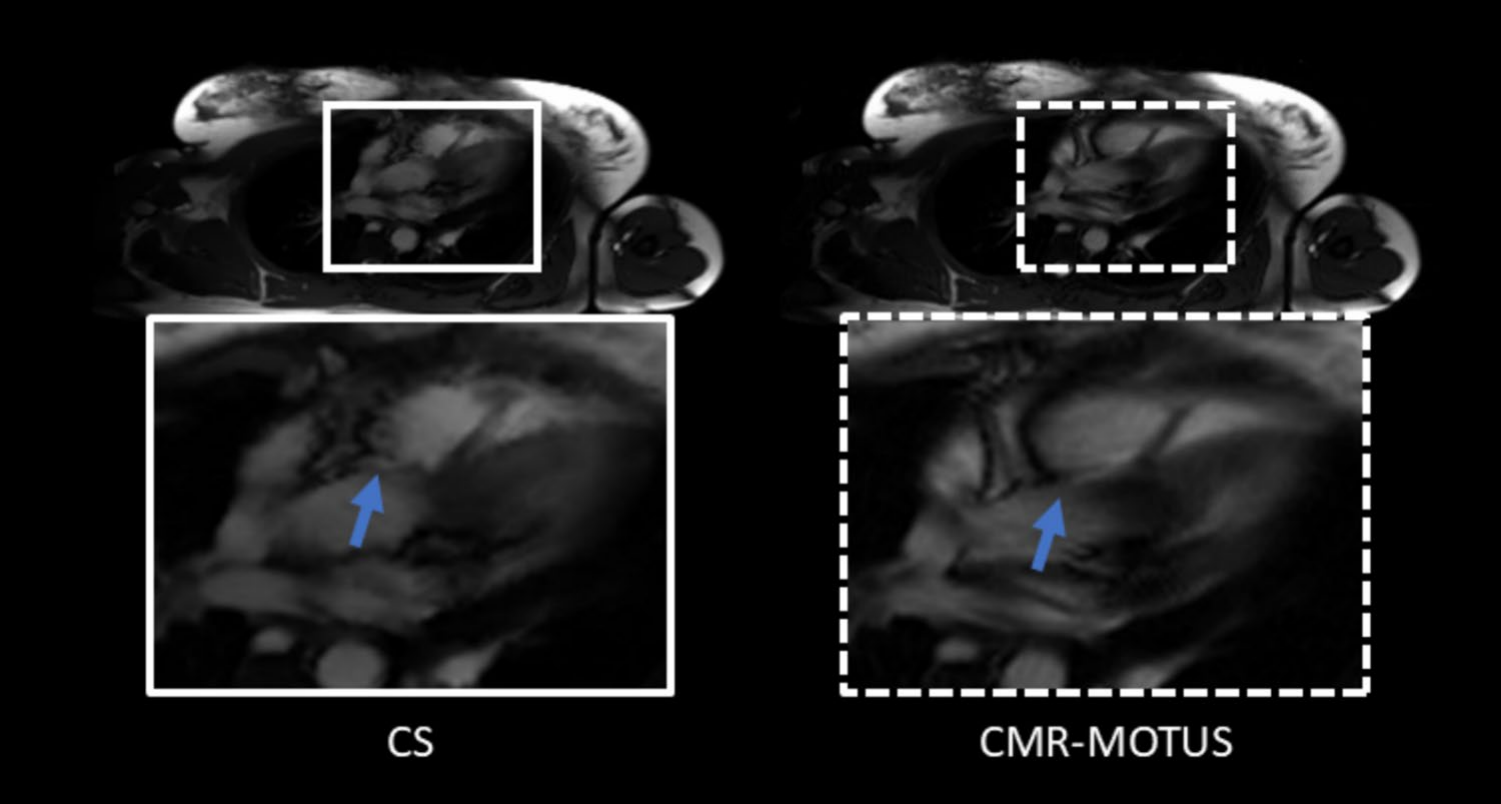
Snapshot from the reconstructed long-axis (LAX) cine images in experiment 2 using compressed sensing (CS) on the left and CMR-MOTUS on the right. Both methods capture the dynamics in the data. The region indicated by the blue arrow on the CS image shows motion-induced blurring due to the limitations of CS in capturing rapid and complex motion dynamics. CS reconstructions tend to introduce temporal blurring in areas with fast-moving structures because they rely on sparsity constraints that may not fully account for intricate motion patterns. On the other hand, CMR-MOTUS is designed to preserve the anatomical details by modelling the motion using motion fields

The LAX views in Fig. 5 show that both methods capture cardiac and respiratory dynamics. However, this specific LAX view and region contains complex motion dynamics and fast-moving thin structures. Temporal blurring becomes more apparent in these regions in the CS cine than in the CMR-MOTUS cine.

**Fig. 6:**
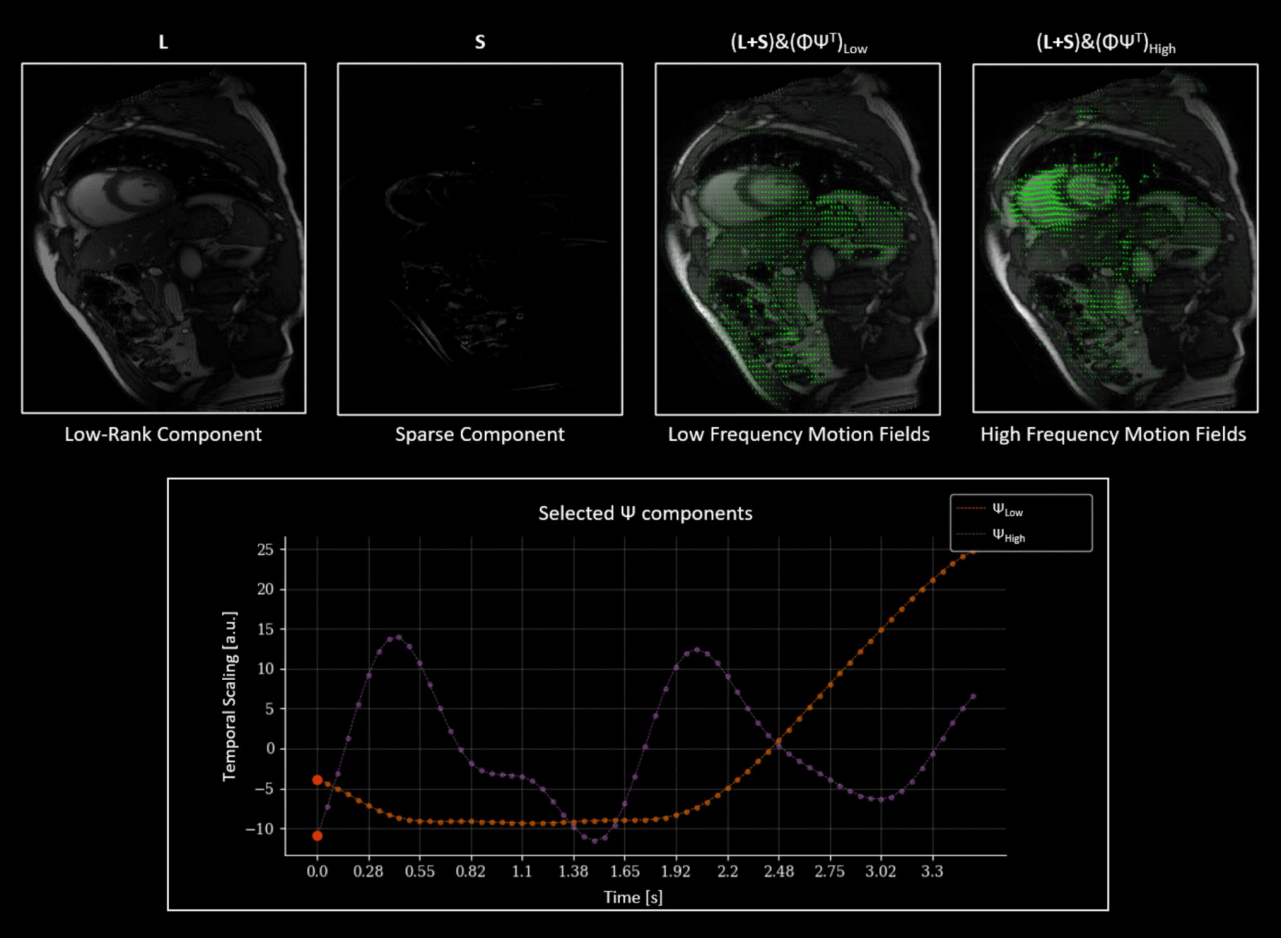
Disentanglement of dynamics in the short axis (SAX) view data in experiment 2 using CMR-MOTUS. Starting above, left to right. This figure is a snapshot at the time point indicated by the red dot in the plot. The figure is available as a video in the supplementary materials. The low-rank component captures the major structures in one motion state. The sparse component captures the local intensity changes, which are mainly due to residual motion, blood flow, and through-plane motion. The low-frequency motion fields are the low-rank motion field components that can be seen to have a low temporal frequency and these are visualized by warping the L + S with the motion fields overlaying. The high-frequency motion Fields are the low-rank Motion Field components that can be seen to have a high temporal frequency, and these are visualized by warping the L + S with overlaying motion fields. The temporal scaling $\Psi$ of two selected components are plotted against time below which shows the clear distinction between a slower $\Psi_{Low}$ and a faster $\Psi_{High}$

Figure 6 highlights the disentanglement of SAX cine dynamics using CMR-MOTUS. These correspond to the low-rank component of the images $({\mathrm{L}})$, the sparse component of the images$({\mathrm{S}})$, and the low-rank motion fields$({\mathrm{D}})$. Combining these components produces the CMR-MOTUS cine $({\mathrm{I}})$. Major anatomical structures are captured in L in one static motion state, whilst S captures local intensity changes, including blood flow through arteries. There is little residual motion captured in S, which can be due to through-plane motion. From the low-rank motion model, it was possible to separate the cardiac motion fields from the respiratory motion fields based on the frequency content of the temporal scaling components (i.e. the vectors in$\Psi$).

### Test 3: in-vivo breath-hold ECG-triggered first-pass myocardial perfusion

**Fig. 7:**
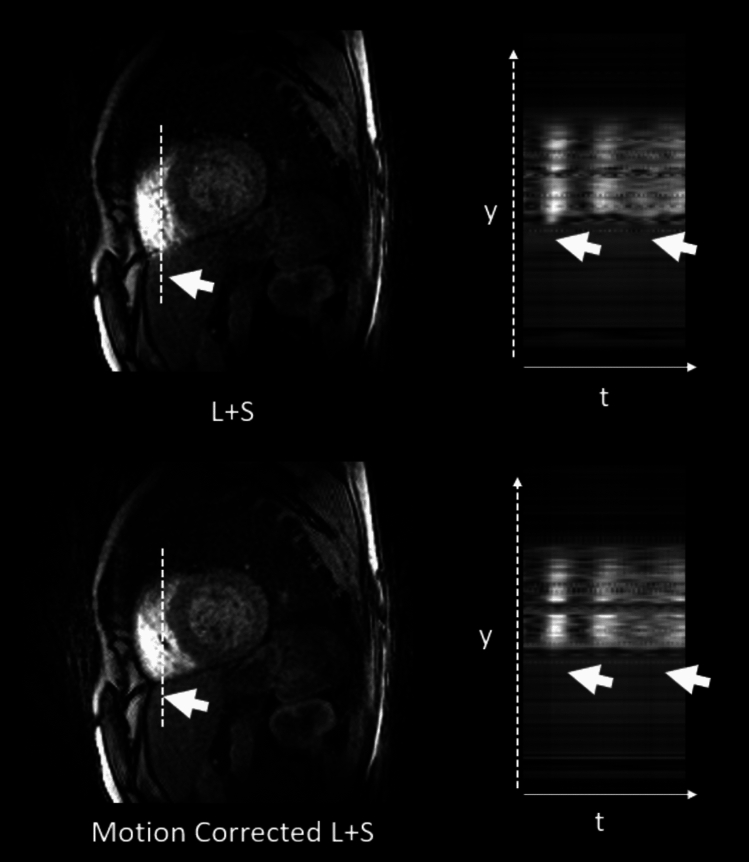
Reconstructions in experiment 3 of a breath hold (BH) ECG-triggered first-pass myocardial perfusion. The top left shows a frame from the ECG-triggered cine using a standard low-rank plus Sparse decomposition (L + S) reconstruction without motion correction. The bottom left shows a frame from the proposed framework (CMR-MOTUS) motion-corrected (L + S) reconstruction using the estimated bulk motion fields. On the right are y–t profiles of the left reconstructions along the dashed lines. Arrows point out the locations of motion corruption due to bulk motion from an imperfect breath hold. This bulk motion is corrected as seen in the profile of the Motion Corrected L + S

The reconstructed cines frame with y–t profile plot are shown in Fig. 7. In the non-corrected L + S images, there are two time points where the bulk motion induces blurring, mainly at the edge of the RV indicated with the arrow. CMR-MOTUS can resolve the underlying bulk motion, which is disentangled from the GBCA inflow. Using these high-quality motion fields, the bulk motion can be motion-corrected for in the L + S reconstruction as seen in the y–t profile plots.

### Test 4: in vivo time-resolved first-pass myocardial perfusion

**Fig. 8:**
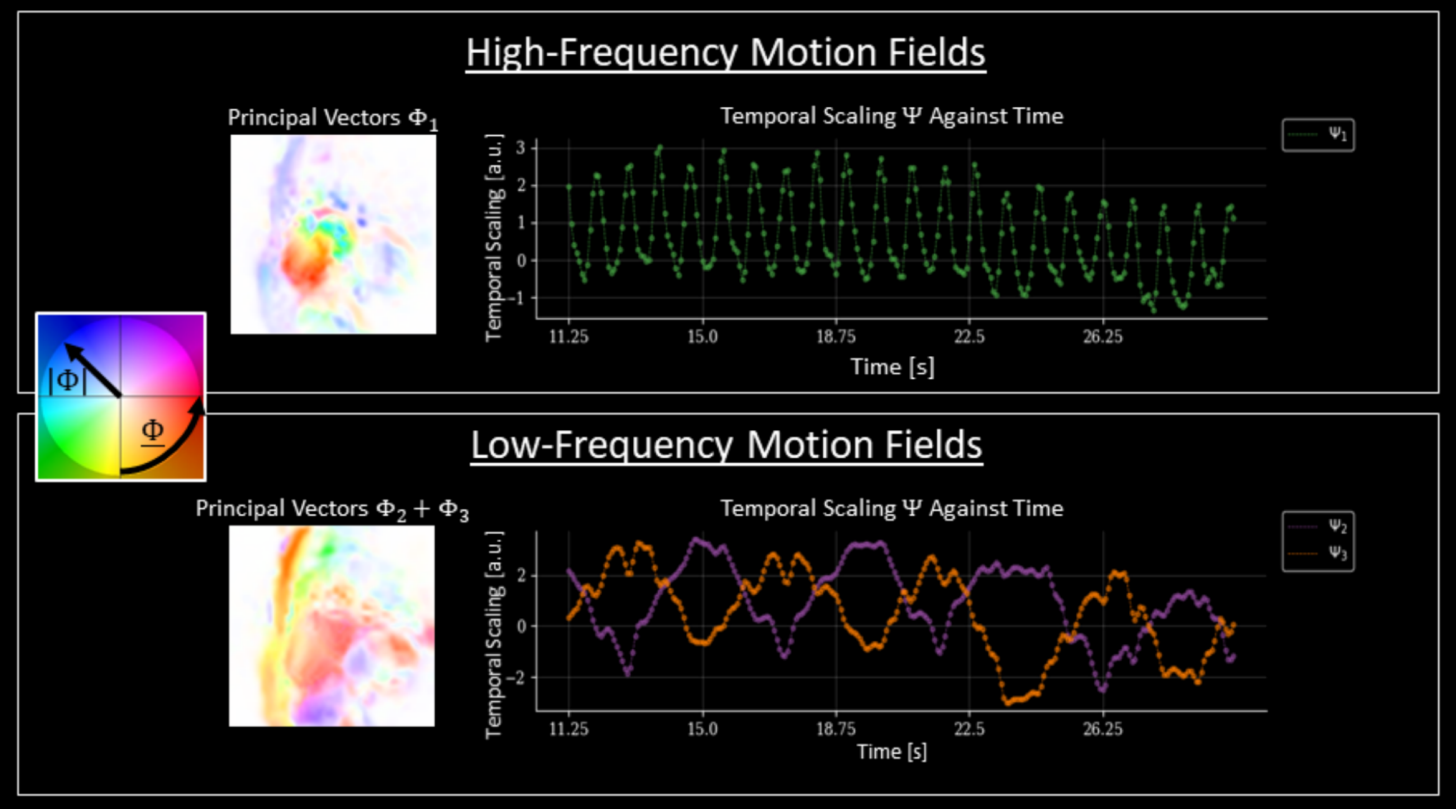
CMR-MOTUS motion field disentanglement using data in experiment 4 of free-breathing first-pass myocardial perfusion without ECG-triggering. Top: the high-frequency motion fields with their principal vectors $\Phi_{1}$ visualized on the left and their temporal component $\Psi_{1}$ plotted against time on the right. Bottom: shows the high-frequency motion fields with their principal vectors $\Phi_{2} + \Phi_{3}$ visualized on the left and their temporal component $\Psi_{2} + \Psi_{3}$ plotted against time on the right

Figure 8 highlights the disentanglement of the motion components. From the low-rank motion model D it was possible to separate different types of motion based on the frequency content of the temporal scaling components $\Psi$ and direction of the principal spatial components $\Phi$. For the high temporal frequency motion fields, the corresponding spatial components exhibit different directions within the myocardium, whilst the low temporal frequency motion fields are mostly aligned along a single direction (feet-head) within the myocardium. This can indicate that the high-frequency motion fields capture cardiac contractions and the low-frequency motion fields capture the almost bulk-like breathing motion.

**Fig. 9:**
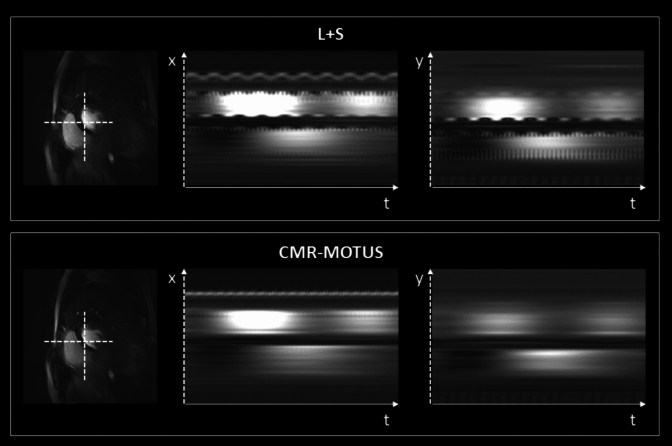
x-t profile plots obtained using data from experiment 4 of free-breathing first-pass myocardial perfusion without ECG-triggering. (Top) Standard low-rank plus sparse decomposition (L + S) reconstruction, x-t and y–t plot. (Bottom) CMR-MOTUS motion corrected L + S reconstruction, x-t and y–t plot (Left) short-axis (SAX) image indicates where the profile was drawn (dashed line). The standard L + S x-t plot is modulated by respiratory and cardiac motion. The proposed L + S x-plot shows that the anatomy is spatially aligned since the motion is corrected for

Leveraging the disentanglement of motion dynamics, a motion-corrected L + S reconstruction was achieved using all the data from the entire acquisition. Figure 9 shows the x–t and y–t plot with and without this motion correction in the L + S reconstruction. Modulations due to respiratory and cardiac motion are seen in the x–t and y–t plot without motion correction. These modulations are not seen in the motion corrected plots, hence giving a motion corrected GBCA inflow.

## Discussion

We presented a multi-scale dynamic decomposition framework to capture cardiac/respiratory motion and contrast dynamics in first-pass myocardial perfusion MRI without ECG-gating and breath holds. In contrast to methods that require ECG triggering of CMR, our approach enabled a more efficient acquisition by using a time-resolved motion model, which correlates all timepoints. This was achieved using a joint reconstruction of high-quality motion fields with the extended MR-MOTUS model and a motion-corrected L + S image model. This combination of frameworks enabled the disentanglement of different motion components, such as respiratory and cardiac motion.

To successfully achieve our goal, we overcame a limitation of the previous MR-MOTUS implementation: the reference image was assumed to be static, meaning no changes in contrast or external signal sources. This assumption fell short in describing mass flowing into the FOV, especially problematic in cardiac MRI examinations, where there is frequent blood inflow and outflow. The proposed contrast time-varying reference image gives more flexibility to the model to describe this mass inflow. The presented results suggest a significant improvement in modeling physiological contrast and motion dynamics.

One common method of mitigating motion artifacts is to discard parts of the data thatcontain bulk motion. However, this might not be feasible in time-sensitive examinations, such as in first-pass myocardial perfusion. This is seen in Fig. 6 (top row, pink arrows) where images of contrast inflow are corrupted due to an imperfect breath hold, which cannot be discarded for perfusion analysis. With CMR-MOTUS, the bulk motion is estimated and is corrected for in the image reconstruction as seen in Fig. 6 (bottom row, pink arrows). Another motion mitigation method triggers scans to a certain phase in the breathing or cardiac cycle with the help of breath holds and ECG, which effectively reduces the efficiency of the scan. However, irregular heart rates impact the temporal resolution negatively, which in turn impacts the downstream task of perfusion quantification [36]. With CMR-MOTUS, motion is mitigated through the estimation of high-quality motion fields to use a free-running continuous data stream as seen in Fig. 9.

Even in the extended model, there is still a chance that motion will be captured by the L + S decomposition of the image time-series ${\boldsymbol{Q}}$. Moreover, contrast variation could be captured by the motion fields in the MR-MOTUS framework which corrupts their quality. As seen in Fig. 6, there is an imperfect separation between these physiological dynamics e.g. respiratory versus cardiac. Instead, we have labelled the components as sparse, low frequency and high frequency components without explicitly attaching a physiological meaning. Although the result shows a promising direction for this type of modelling in the separation of cardiac, respiratory and contrast inflow dynamics. For first-pass perfusion, it is essential to observe the contrast inflow without any motion corruption. Hence, for these examinations the separation between motion and contrast agent inflow dynamics is required. In cases where complete separation is difficult with CMR-MOTUS, fine tuning of the regularization weights and motion field parameters is needed. One improvement would be to further explicitly model the underlying motion and contrast inflow dynamics, such as through sub-space completion [37].

For standard cine imaging, the proposed CMR-MOTUS framework needs thorough clinical validation to confirm the potential in improving diagnosis. The clinical impact that CMR-MOTUS reconstructions have on perfusion analysis was not investigated, as this work focused on the technical feasibility of the framework. The method uses free-running acquisitions, which improves patient comfort and reduces the time necessary to prepare patients since no ECG is required. Additionally, common ECG artifacts such as mis-triggering or irregular heartbeats are avoided. Since the cardiac motion fields could be disentangled from the image reconstruction, they might be valuable for determining myocardial strain. We leave this analysis to future work. Finally, exploring the extension of motion-correcting free-running quantitative mapping sequences would improve the clinical utility of the framework.

## Conclusion

For the few cases considered in this work, CMR-MOTUS offers a significant advancement in capturing cardiac and respiratory motion, as well as contrast dynamics, in first-pass myocardial perfusion MRI without the need for ECG-gating and breath holds. By leveraging a joint-reconstruction approach with the extended MR-MOTUS model and a motion-corrected L + S image model, the framework successfully disentangles different motion and contrast components, providing an efficient and flexible acquisition method.

## Supplementary Information

Below is the link to the electronic supplementary material.

Supplementary file1 (GIF 26714 KB)

Supplementary file2 (GIF 17101 KB)

Supplementary file3 (GIF 15003 KB)

Supplementary file4 (GIF 10934 KB)

Supplementary file5 (PNG 26 KB)

Supplementary file6 (PNG 29 KB)

## Data Availability

The 2D time-resolved k-space data supporting reconstructions in Figs. 4, 5, and 6 are publicly available from [26]. The 2D time-resolved k-space data supporting reconstructions in Figs. 7,8, and 9 are not publicly available to protect patient privacy.
