# Supplementary figures and images for: Free-running time-resolved first-pass myocardial perfusion using a multi-scale dynamics decomposition: CMR-MOTUS
Source: MAGMA. 2025 Sep 23;39(2):173–86. doi: 10.1007/s10334-025-01291-x (PMC13124790; doi:10.1007/s10334-025-01291-x)

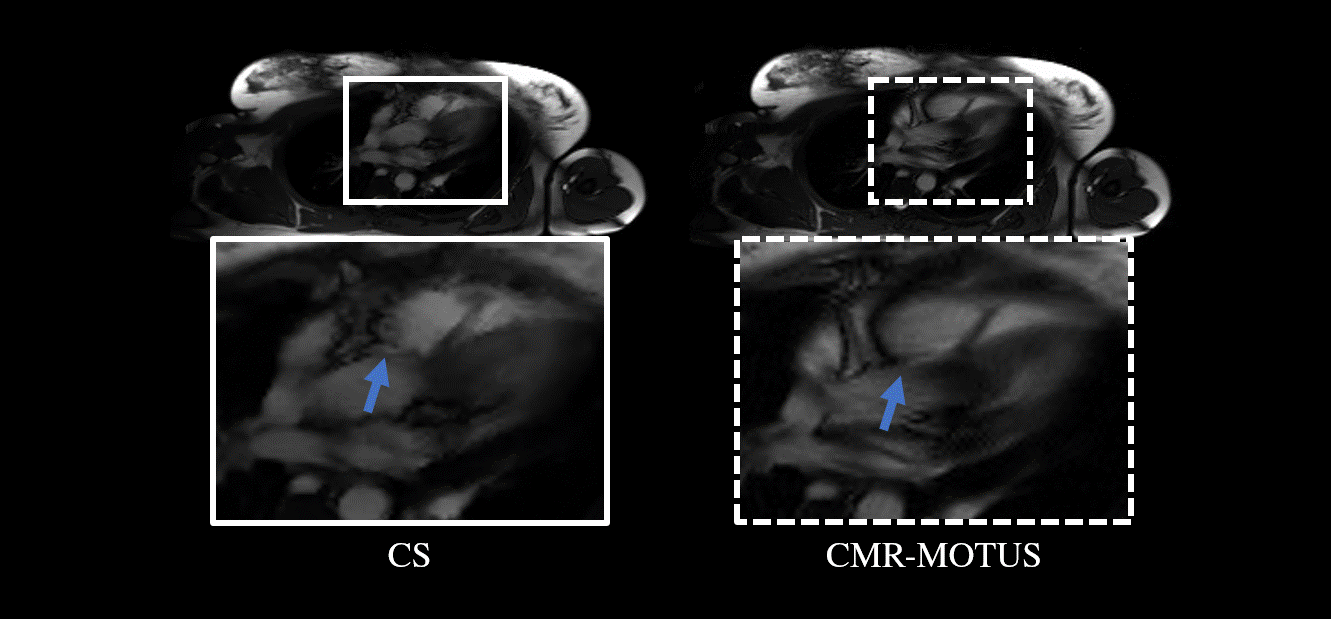

Supplement: Supplementary file 1 — Supplementary file1 (GIF 26714 KB) [file 10334_2025_1291_MOESM1_ESM.gif]

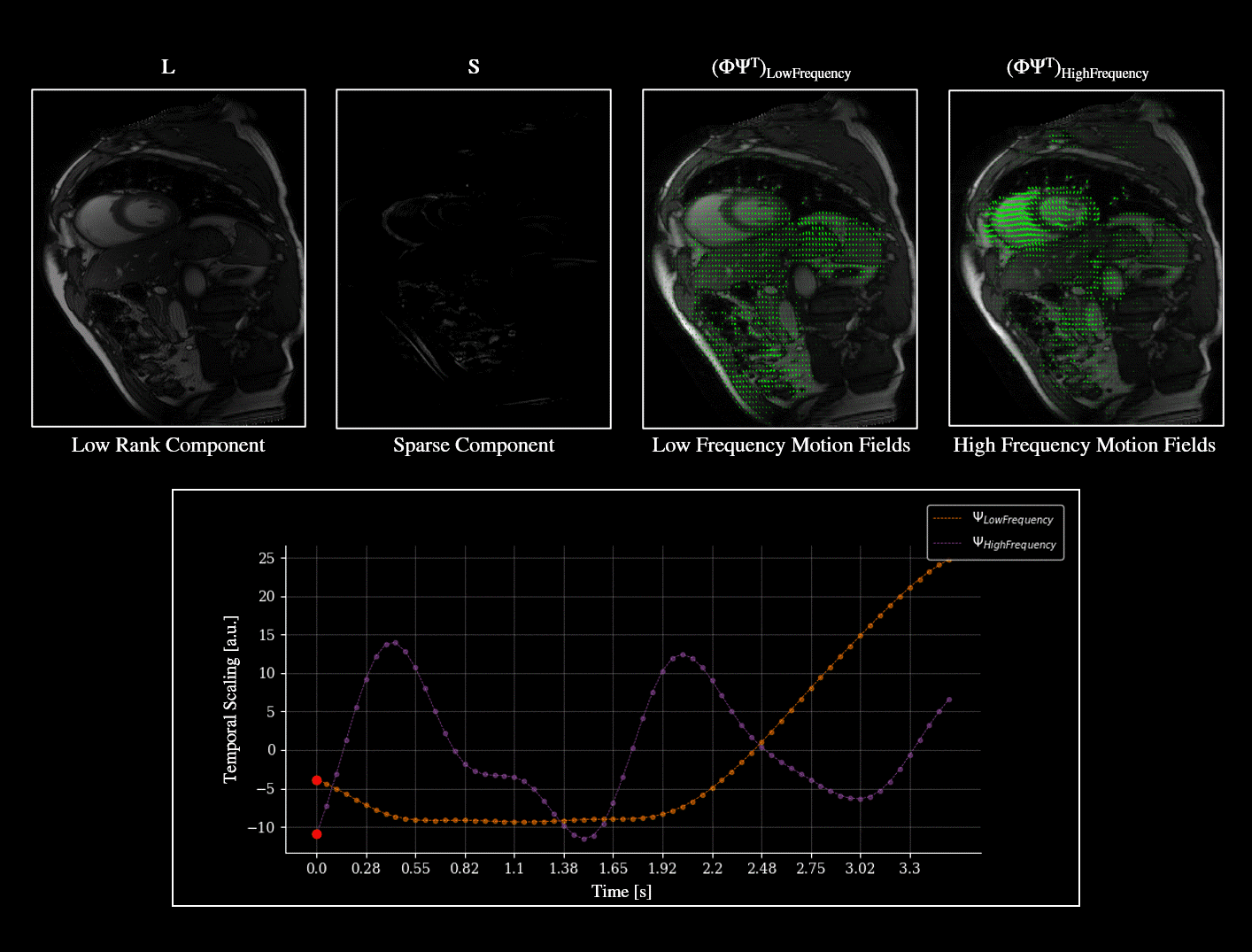

Supplement: Supplementary file 2 — Supplementary file2 (GIF 17101 KB) [file 10334_2025_1291_MOESM2_ESM.gif]

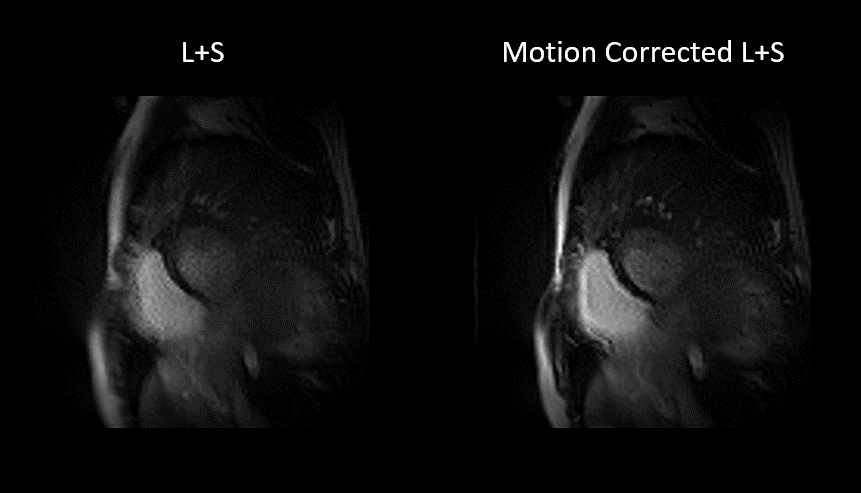

Supplement: Supplementary file 3 — Supplementary file3 (GIF 15003 KB) [file 10334_2025_1291_MOESM3_ESM.gif]

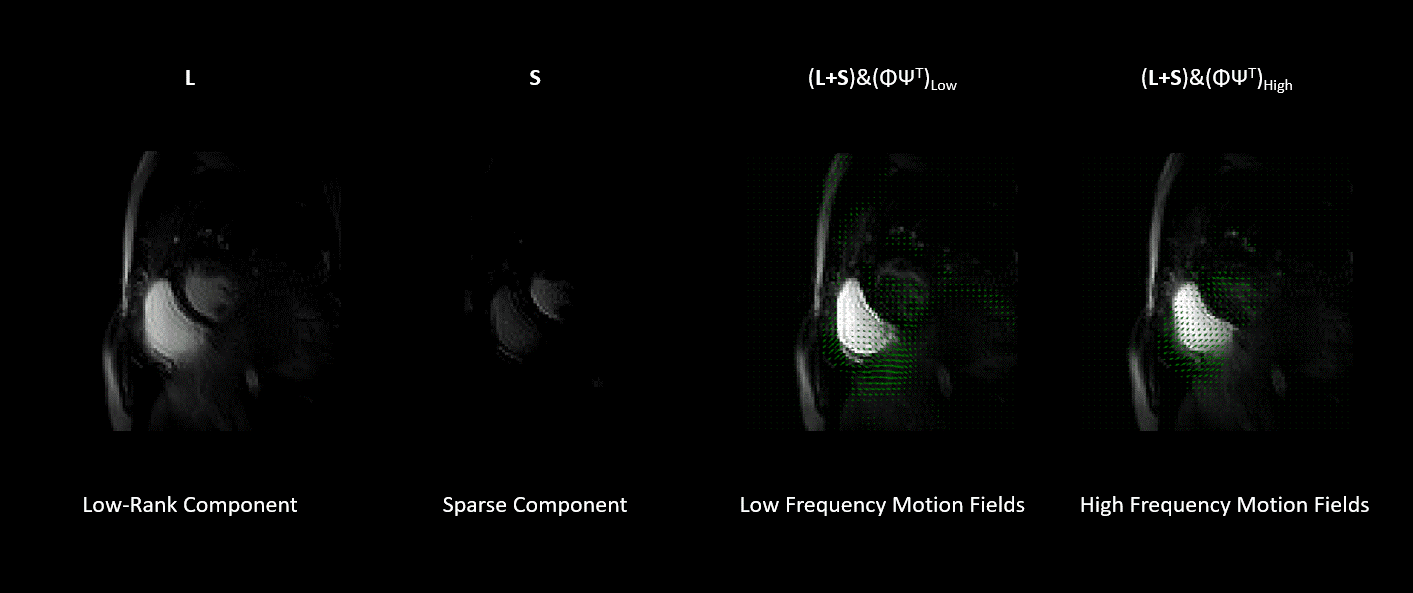

Supplement: Supplementary file 4 — Supplementary file4 (GIF 10934 KB) [file 10334_2025_1291_MOESM4_ESM.gif]

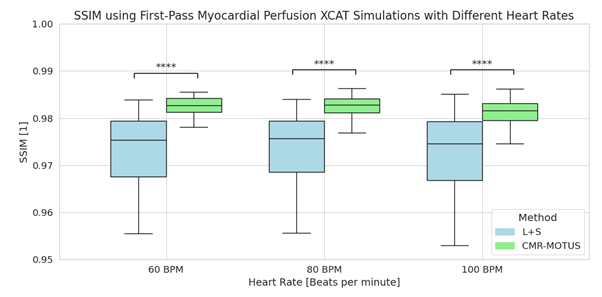

Supplement: Supplementary file 5 — Supplementary file5 (PNG 26 KB) [file 10334_2025_1291_MOESM5_ESM.png]

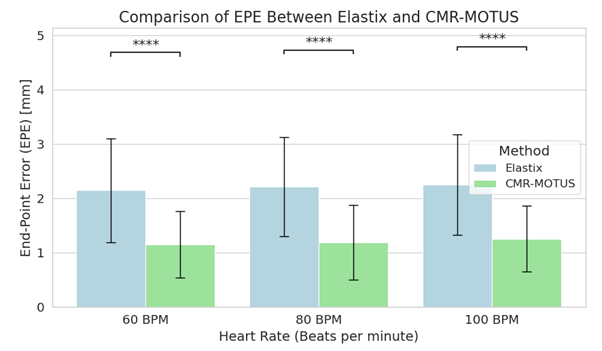

Supplement: Supplementary file 6 — Supplementary file6 (PNG 29 KB) [file 10334_2025_1291_MOESM6_ESM.png]
